# Etymologia: tuberculosis

**DOI:** 10.3201/eid1205.ET1205

**Published:** 2006-05

**Keywords:** etymology, etomologia, tuberculosis, mycobacterium

## [too-ber′′ku-lo′sis]

Any of the infectious diseases of humans or other animals caused by bacteria of the genus *Mycobacterium*. From the Latin *tuberculum*, "small swelling," the diminutive form of *tuber*, "lump." Tuberculosis has existed in humans since antiquity; it is believed to have originated with the first domestication of cattle. Evidence of tuberculosis has been shown in human skeletal remains and mummies from as early as 4000 bc. *Mycobacterium bovis* bacillus Calmette-Guérin has been successfully used to immunize humans since 1921, and treatment (rather than prevention) of tuberculosis has been possible since the introduction of streptomycin in 1946. Hopes of completely eliminating the disease, however, have been diminished since the rise of drug-resistant *M. tuberculosis* strains in the 1980s.

**Sources:** Dorland's illustrated medical dictionary. 30th ed. Philadelphia: Saunders; 2003; Merriam-Webster's collegiate dictionary. 11th ed. Springfield (MA): Merriam-Webster Incorporated; 2003; and http://www.wikipedia.org

